# Supplementary material for: Motivational effectiveness of prosocial public health messaging to reduce respiratory infection risk: a systematic review and meta-analysis
Source: Commun Med (Lond). 2025 Dec 24;6:42. doi: 10.1038/s43856-025-01296-6 (PMC12820126; doi:10.1038/s43856-025-01296-6)
Supplement: Supplementary file 2 — Supplementary Information [file 43856_2025_1296_MOESM2_ESM.pdf]

SUPPLEMENTARY MATERIALS

Contents

Supplementary Table S1. Search Strategies 2
Supplementary Table S2. Inclusion criteria (PICOS) 3
Supplementary Table S3. MINDSPACE checklist 4
Supplementary Note 1. MINDSPACE 4
Supplementary Figure S1. BCTs Taxonomy v1 5
Supplementary Note 2. BCTs Taxonomy v1 5
Supplementary Method. Overview of the MoA Ontology 6
Supplementary Results 1. Analysis by country: 7
- ***USA*** 7
- ***European countries*** 7
- ***Japan*** 7
- ***Turkey*** 7

Supplementary Results 2. Analysis by outcome 7
- ***Social distancing intentions*** 7
- ***Mask wearing intentions*** 8
- ***Hand washing intentions*** 8
- ***Diverse-behavioural intentions*** 8
- ***Actual Behaviours*** 9

Supplementary Table S4. Inter-rater agreement for MINDSPACE Coding (PABAK) 10
Supplementary Note 3. Inter-rater agreement for MINDSPACE Coding 10
Supplementary Table S5. Inter-rater agreement for BCT Coding (PABAK) 11
Supplementary Note 4. Inter-rater agreement for BCT Coding 11
Supplementary Table S6. Mechanisms of Action 12
Supplementary Note 5. Mechanisms of Action 12
Supplementary Table S7. Positively affected behaviour-related outcomes by protect others messages 13
Supplementary Table S8. CNMA effect estimates on social distancing intentions 14
Supplementary Figure S2. Network Diagram for social distancing intentions 14
Supplementary Table S9. CNMA effect estimates on mask wearing intentions 15
Supplementary Figure S3. Network diagram for mask wearing intentions 15
Supplementary Table S10. CNMA effect estimates on handwashing intentions 16
Supplementary Figure S4. Network diagram for handwashing intentions 16
Supplementary Table S11. CNMA effect estimates on diverse-behavioural intentions 17
Supplementary Figure S5. Network diagram for diverse-behavioural intentions 17
Supplementary Table S12. CNMA effect estimates of MINDSPACE contextual influencers on personal protective behavioural intentions (self-focused messages) 18
Supplementary Note 6. CNMAs of MINDSPACE for interventions using self-focused messages 18
Supplementary Table S13. CNMA effect estimates of BCTs on personal protective behavioural intentions (self-focused messages) 18
Supplementary Note 7. CNMAs of BCTs for interventions using self-focused messages 18
Supplementary Figure S6. Certainty of the evidence: Personal protective behavioural intentions 19
Supplementary Figure S7. Certainty of the evidence: Social distancing intentions 20
Supplementary Figure S8. Certainty of the evidence: Mask wearing intentions 21
Supplementary Figure S9. Certainty of the evidence: Handwashing intentions 22
Supplementary Figure S10. Certainty of the evidence: diverse-behavioural intentions 23
Supplementary References 24

Supplementary Table S1. Search strategies

MEDLINE (Ovid) & EMBASE*
1. exp respiratory tract infections/<br>2. exp Coronavirus Infections/ or exp SARS Virus/ or exp Severe Acute Respiratory Syndrome/<br>3. exp Coronavirus/<br>4. exp Influenza A Virus, H1N1 Subtype/ or exp Influenza, Human/<br>5. (covid-19 or coronavir* or corona virus* or covid19 or covid 19 or coronavirus infection* or coronavirus or SARS virus or CoV2 or sarscov2 or Severe Acute Respiratory Syndrome or influenza or flu or H1N1 or Ebola or respiratory infection*).ti,ab,kw.<br>6. 1 or 2 or 3 or 4 or 5<br>7. exp disease outbreaks/ or exp epidemics/ or exp Endemic Diseases/<br>8. (epidemic* or pandemic* or endemic* or disease outbreak*).ti,ab,kw.<br>9. 7 or 8<br>10. (public health message* or hygiene communication or health consult*).ti,ab,kw.<br>11. exp Health Communication/<br>12. communication/ or persuasive communication/ or public health message/ or public health campaign/<br>13. ((health* or prosocial* or moral* or utilitarian or norm* or emotion* or hygiene* or persua* or other-focused or other focused or protect* other*) adj3 (messag* or communicat*)).ti,ab,kw.<br>14. text messaging/ or mass communication/ or mass medium/ or social media/<br>15. (mass media or leaflet* or poster* or message framing or text messag* or SMS or social media). ti,ab,kw.<br>16. 10 or 11 or 12 or 13 or 14 or 15<br>17. social behavior/ or altruism/ or social distance/ or social isolation/<br>18. Empathy/<br>19. Masks/<br>20. (face covering* or mask* or hand hygiene or hand wash* or social distanc* or social isolation).ti,ab,kw.<br>21. exp Hand Hygiene/<br>22. (prosocial* behavior* or pro-social* behaviour* or prosociality or pro-sociality or moral behaviour* or moral behavior* or utilitarian behaviour* or utilitarian behavior* or altruism or empathy).ti,ab,kw.<br>23. (famil* or community or father* or mother*or parent* or home* or friend* or loved one* or elder* or vulnerable* or other*).ti,ab,kw.<br>24. Health Risk Behaviors/<br>25. ((behaviour* or behavio?r*) adj5 (change or influenc* or alter* or modif*)).ti,ab,kw.<br>26. 17 or 18 or 19 or 20 or 21 or 22 or 23 or 24 or 25<br>27. 6 and 9 and 16 and 26

**Although the controlled vocabulary search terms for MEDLINE (MeSH) and EMBASE are not identical, and search strategies typically need to be customized for each database, we ultimately used the same search strategy for both databases in our review as more appropriate.*

PsycINFO
1. exp Respiratory Tract Disorders/ or exp Infectious Disorders/<br>2. (covid-19 or coronavir* or corona virus* or covid19 or covid 19 or coronavirus infection* or coronavirus or SARS virus or CoV2 or sarscov2 or Severe Acute Respiratory Syndrome or influenza or flu or H1N1 or Ebola or respiratory infection*).mp.<br>3. exp Coronavirus/<br>4. exp Influenza/<br>5. 1 or 2 or 3 or 4<br>6. exp Disease Outbreaks/<br>7. (epidemic* or pandemic* or endemic* or disease outbreak*).mp.<br>8. 6 or 7<br>9. exp Communications Media/<br>10. (public health message* or hygiene communication or health consult*).mp.<br>11. exp Mass Media/ or exp Messages/<br>12. communication/ or persuasive communication/ or public health message/ or public health campaign/<br>13. ((health* or prosocial* or moral* or utilitarian or norm* or emotion* or hygiene* or persua* or other-focused or other focused or protect* other*) adj3 (messag* or communicat*)).mp.<br>14. text messaging/ or mass medium/ or mass communication/ or social media/<br>15. 9 or 10 or 11 or 12 or 13 or 14<br>16. social behavior/ or altruism/ or social distance/ or social isolation/<br>17. Empathy/<br>18. ((behaviour* or behavio?r*) adj5 (change or influenc* or alter* or modif*)).mp.<br>19. (face covering* or mask* or hand hygiene or hand wash* or social distanc* or social isolation).mp.<br>20. (prosocial* behavior* or pro-social* behaviour* or prosociality or pro-sociality or moral behaviour* or moral behavior* or utilitarian behaviour* or utilitarian behavior* or altruism or empathy).mp.<br>21. (famil* or community or father* or mother*or parent* or home* or friend* or loved one* or elder* or vulnerable* or other*).mp.<br>22. exp Health Behavior/<br>23. 16 or 17 or 18 or 19 or 20 or 21 or 22<br>24. 5 and 8 and 15 and 23

Scopus
TITLE-ABS-KEY(“respiratory infection*” OR influenza OR Coronavirus OR “*SARS Virus” OR covid-19 OR coronavir* OR “corona virus*” OR covid19 OR “covid 19” OR “coronavirus infection*” OR CoV2 OR sarscov2 OR “Severe Acute Respiratory Syndrome” OR influenza OR flu OR H1N1 OR Ebola)<br>AND<br>TITLE-ABS-KEY(Pandemic* OR “disease outbreak*” OR epidemic* OR “Endemic Disease*”)<br>AND<br>TITLE-ABS-KEY((“public health messag*” OR “hygiene communication” OR “health consult*” OR “*Health Communication” OR communicat* OR “persuasive communication” OR “public health campaign*”) OR (“prosocial messag*” OR “moral messag*” “norm-based messag*” OR “other-focused messag*” OR “other focused messag*” OR “protect* other*”) OR (“text messag*” OR “mass communication” OR “social media” OR “mass media”))<br>AND<br>TITLE-ABS-KEY((“social behavio*” OR altruis* OR “social distanc*” OR “social isolation”) OR (Empath* OR prosociality OR pro-sociality OR “prosocial* behavio*”) OR (mask OR “face covering” OR “Hand adj1 wash*”) OR (“Health Risk Behavio*” OR “behavio* change” OR “alter behavio” OR “behavio* influenc*”))

Supplementary Table S2. Inclusion criteria (PICOS)

Population:	General public including population with or without vulnerabilities and/or people who are/are not already infected. There were no restrictions placed on geographical region.
Interventions:	Interventions focused on communication via mass media, social media or print media (such as leaflets and posters) or health professional advice via consultation where messages about protecting others are included. Prosocial messages are defined those that refer to benefits of others, whether or not they involve an overall cost to self, and include a variety of important social behaviours such as helping, sharing and cooperation.[1] A prosocial (protect-others) message was referred either to (a) loved ones (friends and family), or (b) members of the public (e.g. country; community; vulnerable members of community or country with weakened immune systems such as elderly and chronically ill; keyworkers), or (c) loved ones or public with the addition of self.
Comparisons:	No message or active control with messages focused on self-protection or messages that contain no motivational content.
Outcomes:	Any behaviour relevant to reducing transmission of respiratory infections (e.g., socializing, hand hygiene, social distancing, face masks, touching face, catching coughs/sneezes, testing, reporting contacts, isolating if infected).
Study Design:	RCTs and quasi experimental studies (such as controlled before-an-after studies, interrupted time series at multiple time points).

Supplementary Table S3. MINDSPACE checklist

Contextual influencer	Behaviour	Examples of MINDSPACE checklist for designing healthcare behaviour change interventions.
Messenger	Who communicates information matters, especially in terms of the perceived authority (whether formal or informal) of the messenger	For example, demographic and behavioural similarities between the expert and the audience can improve the effectiveness of the intervention. We are also affected by the feelings we have for the messenger. In general, public health authorities (e.g. CDC) and medical professionals are the most trusted messengers during a public health emergency[2]
Incentives	Human evaluations of outcomes (benefits and costs) are shaped by specific mental processes (known as heuristics and biases) such as loss aversion (strongly avoiding losses), overweighting small probabilities, present bias, and reference points	The intuition behind incentives during a pandemic is that interventions that increase the cost of activities that are high-transmission risk, decrease the benefit of doing these activities (e.g., bans on large gatherings, closure of entertainment venues), or decrease the cost of not doing them (e.g., paid sick leave) will encourage proper social isolation.[2]
Norms	Individuals are strongly influenced by what others do, which shape the social and cultural norms in a society or social network	For example, Instagram users were able to add a "Stay Home" sticker to any of their stories, demonstrating their support for staying home to their social networks.[2]
Defaults	In many situations, individuals rely on default options, the options that are preselected if an individual does not make an active choice	For example, to encourage social distancing, video conferences (such as Skype, Zoom, Microsoft Teams) or phone calls replaced face-to-face meetings as the new default.[2]
Salience	individuals' behaviours are influenced by what draws attention, what is novel, and seems relevant.	For example, an image to promote the salience of engaging in social distancing and handwashing behaviours or appeals to local culture can increase the salience of messages.[2]
Priming	Individuals are often subconsciously influenced by situational cues (such as images, words, sounds, sensations) that activate, or prime, information in their memory.	For example, priming prosocial behaviour, such as social distancing, can be achieved through visual aids, by placing brightly coloured tape lines on the floor in grocery stores to help people maintain distance between other shoppers.[2]
Affect	Emotional associations can powerfully shape individuals' actions, with good moods leading to unrealistically optimistic judgments and bad moods leading to unrealistically pessimistic judgments.	For example, one nudge to encourage connection while staying physically separated was rebranding the phrase "social distancing" as "physical distancing".[2]
Commitments	They can help achievement of long-term goals as they are linked to our public promises (statements, pledges) and also to reciprocity through a desire for fairness	For example, Facebook users could add a ''Stay At Home'' frame to their profile photo to encourage self-isolation.[2]
Ego	Individuals tend to behave in ways that support the impression of a positive and consistent self-image	Messages that emphasize protection of others (e.g., ''others'' are more likely to get sick) and deontological messages (duties to protect others) can motivate behaviour change.[2]

Research source: Dolan P, Hallsworth M, Halpern D, King D, Metcalfe R, Vlaev I. Journal of Economic Psychology 2012; 33(1): 264-77[3]

Supplementary Note 1. MINDSPACE checklist

The MINDSPACE checklist[3], which stands for the above nine contextual influencers, is associated with ideas about *nudge*[4] and is based on the theory that much behaviour is automatic (operated largely through automatic neurobiological systems and psychological processes) and can be influenced by the context in which decisions are made[5]. A nudge is defined as "any aspect of choice architecture that alters people's behaviour in a predictable way without forbidding any options"[4]. It also refers to generalisations about human behaviour, cognition or emotion that intervention designers can use to make their interventions more effective[6].

MINDSPACE contextual influencers were double-coded by reviewers using a MINDSPACE extraction form. The contextual influences were coded separately for intervention and control groups. Disagreements between reviewers were discussed and reconciled.

Supplementary Figure S1. BCTs Taxonomy v1

Grouping and BCTs

1. Goals and planning

1.1. Goal setting (behaviour)
1.2. Problem solving
1.3. Goal setting (outcome)
1.4. Action planning
1.5. Review behaviour goal(s)
1.6. Discrepancy between current behaviour and goal
1.7. Review outcome goal(s)
1.8. Behavioural contract
1.9. Commitment

2. Feedback and monitoring

2.1. Monitoring of behaviour by others without feedback
2.2. Feedback on behaviour
2.3. Self-monitoring of behaviour
2.4. Self-monitoring outcome(s) of behaviour
2.5. Monitoring of outcome(s) of behaviour without feedback
2.6. Biofeedback
2.7. Feedback on outcome(s) of behaviour

3. Social support

3.1. Social support (unspecified)
3.2. Social support (practical)
3.3. Social support (emotional)

4. Shaping knowledge

4.1. Instruction on how to perform the behaviour
4.2. Information about Antecedents
4.3. Re-attribution
4.4. Behavioural experiments

5. Natural consequences

5.1. Information about health consequences
5.2. Salience of consequences
5.3. Information about social and environmental consequences
5.4. Monitoring of emotional consequences
5.5. Anticipated regret
5.6. Information about emotional consequences

6. Comparison of behaviour

6.1. Demonstration of the behaviour
6.2. Social comparison
6.3. Information about others' approval

7. Associations

7.1. Prompts/cues
7.2. Cue signalling reward
7.3. Reduce prompts/cues
7.4. Remove access to the reward
7.5. Remove aversive stimulus
7.6. Satiation
7.7. Exposure
7.8. Associative learning

8. Repetition and substitution

8.1. Behavioural practice/rehearsal
8.2. Behaviour substitution
8.3. Habit formation
8.4. Habit reversal
8.5. Overcorrection
8.6. Generalisation of target behaviour
8.7. Graded tasks

9. Comparison of outcomes

9.1. Credible source
9.2. Pros and cons
9.3. Comparative imagining of future outcomes

10. Reward and threat

10.1. Material incentive (behaviour)
10.2. Material reward (behaviour)
10.3. Non-specific reward
10.4. Social reward
10.5. Social incentive
10.6. Non-specific incentive
10.7. Self-incentive
10.8. Incentive (outcome)
10.9. Self-reward
10.10. Reward (outcome)
10.11. Future punishment

11. Regulation

11.1. Pharmacological support
11.2. Reduce negative emotions
11.3. Conserving mental resources
11.4. Paradoxical instructions

12. Antecedents

12.1. Restructuring the physical environment
12.2. Restructuring the social environment
12.3. Avoidance/reducing exposure to cues for the behaviour
12.4. Distraction
12.5. Adding objects to the environment
12.6. Body changes

13. Identity

13.1. Identification of self as role model
13.2. Framing/reframing
13.3. Incompatible beliefs
13.4. Valued self-identify
13.5. Identity associated with changed behaviour

14. Scheduled consequences

14.1. Behaviour cost
14.2. Punishment
14.3. Remove reward
14.4. Reward approximation
14.5. Rewarding completion
14.6. Situation-specific reward
14.7. Reward incompatible behaviour
14.8. Reward alternative behaviour
14.9. Reduce reward frequency
14.10. Remove punishment

15. Self-belief

15.1. Verbal persuasion about capability
15.2. Mental rehearsal of successful performance
15.3. Focus on past success
15.4. Self-talk

16. Covert learning

16.1. Imaginary punishment
16.2. Imaginary reward
16.3. Vicarious consequences

Reference source: Michie S, Richardson M, Johnston M, et al. Annals of behavioral medicine 2013; 46(1): 81-95[7]

Supplementary Note 2. BCTs Taxonomy v1

The BCTT encompasses techniques that have been proven to influence behaviour through various psychological mechanisms (both reflective and automatic).[8,9] A Behaviour Change Technique (BCT) is defined as an irreducible, observable and replicable component designed to change behaviour. It is the smallest component of an intervention designed to alter or redirect causal processes and can be used alone or in combination with other BCTs.[8,10] The BCTT has been mainly used to identify, through systematic reviews, the presence of BCTs that are more frequently used and/or more effective across a wide range of behaviour change interventions in diverse populations.[11]

BCTs were double-coded by reviewers using the Behaviour Change Techniques Taxonomy v1[7] that provides a list of 93 clearly labelled and defined BCTs, organised in 16 higher-order groupings (Goals and planning; Feedback and monitoring; Social support; Shaping knowledge; Natural consequences; Comparison of behaviour; Associations; Repetition and substitution; Comparison of outcomes; Reward and threat; Regulation; Antecedents; Identity; Scheduled consequences; Self-belief; Covert learning). The reviewers followed the guidelines adapted from the BCTTv1 online training website (www.bct-taxonomy.com). BCTs were coded separately for intervention and control groups, using a BCT extraction form. Disagreements between reviewers were discussed and reconciled. The reliability of coding of BCTs and MINDSPACE checklist was assessed using the prevalence and bias-adjusted kappa (PABAK) statistic.[12] PABAK was used because it adjusts for shared bias in the coders' use of categories and high prevalence of negative agreement (e.g., when both coders agree that codes are absent).

Supplementary Method. Overview of the MoA Ontology

The MoA Ontology (Table D1, Supplementary Data 1) is being developed for the Human Behaviour-Change Project (HBCP)[13,14] and consists of 261 MoAs that include different classes of concepts, organised in seven hierarchical levels (Level 1 to Level 7). It provides a common conceptual framework for Psychology, allowing to integrate results from different studies. The MoAs are defined as "process that mediate the effect of the intervention on the behavioural outcome"[13] (e.g., BCTs engaging specific psychological processes such as knowledge, beliefs, attitudes, goals, plans, emotions, and habits). The procedure to code the MoAs followed the guidelines provided by the Ontology developers (https://osf.io/um7w6). These specify that to be coded as a MoA, the variable should be measured, and the measurement should be done after the intervention was applied. The MoAs should be coded if they are targeting any behavioural outcome. The papers identified in the review were manually coded according to the guidelines. The MoA was coded even if the article did not explicitly report the link between the intervention and the MoA, and the link between the MoA and the behaviour. All articles were coded independently by two reviewers. Multiple meetings were carried out for training and clarification of terms with the present of an expert who provided consultancy. Finally, disagreements between reviewers were discussed and reconciled.

Supplementary Results 1. Analysis by country

USA

Seventeen of the RCTs[15-21,22, Study 2 & 3,23, Study 1 & 2,24, Study 1 & 2] reporting positive effects regarding the included messages, focusing on the "protect-others" principle, were conducted in the USA. Additionally, one RCT[25] reported negative effects, while six RCTs reported no significant differences.[23, Study 3,24, Study 3,26-32] Among the nine RCTs related to social distancing intentions, a significant effect of "protect-others" messages on beliefs about social distancing intentions was observed. Furthermore, three RCTs indicated a significant impact of these messages on the intention to wear face coverings and the motivation to do so. Moreover, one RCT demonstrated a significant effect on hand washing rates, intentions, adherence to hand hygiene, and the use of hand hygiene products. Seven RCTs found significant effects on diverse-behavioural intentions, including contact-avoidance intentions and willingness to engage in protective behaviours. Lastly, three RCTs indicated significant effects on positive outcomes such as happiness, meaningfulness, empathy, social connectedness, contact-avoidance behaviour, infection-prevention behaviour, and hand hygiene behaviour. Only four RCTs revealed significant predictors of personal protective behavioural intentions and actual behaviours concerning respiratory infections. Older age, non-male gender identity, and less secure employment correlated with heightened intentions for COVID-19 preventative measures and wearing face coverings, whereas identifying as White or White/Indigenous was linked to diminished intentions for these measures.

European countries

Five of the RCTs[33-36] reporting positive effects regarding the included messages, focusing on the "protect-others" principle, were conducted in Europe countries, particularly in the UK, Denmark and Germany. Additionally, two RCTs reported no significant differences. Two RCTs found a significant effect of "protect-others" messages on beliefs about social distancing intentions. Furthermore, one RCT indicated a significant impact of these messages on the intention to wear face coverings, while two RCTs demonstrated a significant effect on hand washing rates, intentions, adherence to hand hygiene, and the use of hand hygiene products. Only one RCT revealed significant predictors of personal protective behavioural intentions and actual behaviours concerning respiratory infections. Individuals with poorer health conditions and higher infection risks are most influenced by messages emphasizing the protection of others, particularly their families, while those in better health, facing lower infection risks, and frequently leaving their homes are less affected by such messages.

Japan

Four of the RCTs[37,38] reporting positive effects regarding the included messages, focusing on the "protect-others" principle, were conducted in Asia, particularly in Japan. The RCTs found significant effects on diverse-behavioural intentions, including contact-avoidance intentions and willingness to engage in protective behaviours.

Turkey

One RCT[22, Study 1] that reported positive effects regarding the included messages, focusing on the "protect-others" principle, was conducted in Turkey which is lying partly in Asia and partly in Europe. The RCT found a significant effect of "protect-others" messages on beliefs about social distancing intentions.

Supplementary Results 2. Analysis by outcome

Social distancing intentions

Overall, the 19 RCTs pertaining to social distancing intentions had positive results, with 11 RCTs reporting positive effects and eight RCTs reporting no difference. The 11 RTCs found significant effect of protect-others messages on the beliefs about social distancing, social distance intentions, the motivation to adhere to physical distancing, persuasiveness to self-isolate, avoid social gathering, stay home and keep a physical distance with others. According to Bokemper, Huber [15] Study 1, two prosocial messages appear particularly promising compared to the control message. The prosocial messages ("Other-regarding, Linear Cooperation" and "Reframing Bravery") increased both outcomes, beliefs about social distancing and social distance intentions. Also, in Study 2 the same prosocial messages appear to be more effective than the control message and a generic interventional arm (the Baseline Informational message) regarding beliefs about social distancing and social distance intentions. Browning, Moss [16] showed that the prosocial message (altruistic goal-orientation) led to significantly greater reports of intention to avoid social gathering and stay home than control (self-protective) message. Ceylan and Hayran [22] found in their three RCTs that participants perceived the prosocial message as more convincing (persuasive) to self-isolate and stay at home than the control message (self-interested message). Similar results were found by Luttrell and Petty [20] (in all three RCTs) where the prosocial message was more persuasive to practice social distancing than the control (self-focused) message, especially when public health moralization increases(to what extent do the participants think that public health is a moral issue). Likewise, in

Study 2 the combined message was also more persuasive than the control (self-focused) message, as moralization increases. However, there were no significant differences between the prosocial message and the combined message (prosocial and self-interested). In addition, Falco and Zaccagni [33] indicated that the message framed with respect to consequences for one's family significantly increased participants' intentions to stay home compared to the control group. Pfattheicher, Nockur [39] also found that the motivation to adhere to physical distancing increased significantly when empathy was induced. In particular, the prosocial ("information + empathy") condition had a significantly higher mean compared with the control condition and the generic intervention condition (information-only). Although Frias-Navarro, Pascual-Soler [29] found no differences between deontological, utilitarian messages and control message, the results show that participants who read the message with deontological, utilitarian content obtained higher scores on the variables of probability of others' washing their hands and the probability of others' staying at home and avoiding social contact, in comparison with those who received the ethical virtue-based message.

Mask wearing intentions

Three RCTs found significant effect of protect-others messages on the intention to wear a face covering and the motivation to wear a mask. According to Bokemper, Huber [15], the prosocial messages ("Reframing Bravery" and "Other-regarding, Linear Cooperation") are associated with a statistically significant increase in mask wearing and appear to be more effective than control message and the generic message (Baseline Information). Capraro and Barcelo [17] found that the prosocial message focused on public ("Your community") gives rise to greater intentions to wear masks than the control. Furthermore, Pfattheicher, Nockur [39] also indicated that the motivation to wear a mask was significantly higher in the prosocial message ("empathy") compared with the control and the generic message (information-only).

Hand washing intentions

Of the 7 RCTs reporting on handwashing intentions, 3 RCTs reported significant positive effects, while 4 RCTs reported no significant difference between intervention and control group. In particular, the 3 positive RCTs found significant effect of protect-others messages on hand washing rates, hand washing intentions, hand-hygiene adherence and hand-hygiene product use. Browning, Moss [16] showed that the prosocial message (altruistic goal-orientation) led to significantly greater reports of intention to wash hands than control (self-protective) message. In Miller, Yardley [34] study, participants who received the prosocial message (had stronger intentions to increase hand washing than those in control. According to Yardley, Miller [36], participants given access to a Web-based intervention (4 sessions of tailored motivational messages and self-regulation support) had higher levels of reported hand-washing intentions for frequent hand-washing in the future than those in the control group (with or without baseline measurement). Hand-washing intentions in the intervention group were higher at 4 weeks than in the control group and remained higher at 12 weeks.

Diverse-behavioural intentions

Overall, the 16 RCTs pertaining to diverse-behavioural intentions had positive results, with 11 RCTs reporting positive effects and 5 RCTs reporting no difference. The 11 RCTs found significant effect of protect-others messages on diverse-behavioural intentions such as contact-avoidance intentions, protective behaviour willingness. In particular, Ceylan and Hayran [22] found in their three RCTs that participants in the prosocial message condition were more motivated to help others than those in the control (self-interested message) condition. Gillman, Iles [18] indicated that prosocial messages (with value and health affirmation) led to higher behavioural intentions and greater willingness to engage in protective behaviours for participants with one or multiple risk factors for COVID-19 who viewed the other-focused messages compared to control (self-focused) message. Miyajima and Murakami [37] found in both RCTs that prevention intention, as well as social desirability, in all three treatment conditions (personal, public and personal + public (Study 1) or family (Study 2)) were significantly higher than in the control condition. However, there were no significant differences between the three treatment conditions. Similarly, Pink, Stagnaro [24] found in Study 1 that compared to the control, four messages (compassion and empathy message, appeal to experts' message, identifiable victim message (yourself) and the active control message) caused an increase in intentions to comply with public health guidelines. In Study 2 the most persuasive message to engage with preventive behaviours was the principle of reciprocity message, which was significantly more persuasive than the control. In addition, Jordan, Yoeli [23] showed in Study 1 and 2 that participants in all treatment conditions (personal, public, personal + public) report directionally higher prevention intentions (e.g., wear a mask when leave the house, completely avoid any unnecessary physical contact with others) than subjects in the control condition. Furthermore, the "public treatment" had larger effect on prevention intentions than the personal treatment. Sasaki, Kurokawa [38] implied that that the prosocial messages (gain-framed altruistic and the loss-framed altruistic) increase the contact-avoidance intention in those who went out more frequently. However, the effect was short-term and disappeared in the third and fourth waves of the pandemic.

Actual Behaviours

Overall, the 7 RCTs pertaining to behaviours had positive results, with 5 RCTs reporting positive effects, one RCT reporting negative effect and one RCT reporting no difference. The 5 RCTs found significant effect of protect-others messages on positive outcomes (such as happiness, meaningfulness, empathy, social connectedness) contact-avoidance behaviour, infection-prevention behaviour and hand hygiene behaviour. Grant and Hofmann [19] study 1 showed a significant increase from pre-test to post-test in the amount of hand-hygiene product used from dispensers with the patient-consequences sign. Although there were no significant differences between conditions in the pre-test use of hand hygiene product, in the post-test, the amount of hand-hygiene product used from dispensers with the patient-consequences sign was significantly greater than the amount used from dispensers with the personal-consequences sign or the control sign. Study 2 showed that hand-hygiene adherence increased significantly on units with the patient-consequences sign. Although during the pre-test period, the units assigned to the personal- and patient- consequences conditions did not differ significantly in hand hygiene adherence, during the post-test period, hand-hygiene adherence was significantly greater on units with the patient-consequences sign than on units with the personal-consequences sign. Sasaki, Kurokawa [38] implied that the prosocial message (gain-framed altruistic) further promotes contact-avoidance behaviour (e.g., decrease frequency of going out to cafes and using public transportation), as well as decreases the level of infection-prevention behaviour (e.g., avoid touching your face, wear a mask when talking) compared to the control group, between the first and the second wave. However, the effect was short-term and disappeared in the third and fourth waves. In addition, Varma, Chen [21] suggested that prosocial behaviour (purchase reusable face masks or hand sanitizer for a needy child from a low-income American family), urged by a prosocial message, enhanced participants' positive psychological outcomes (such as happiness, perceived meaningfulness, positive impact, empathy and social connectedness) and positive affect as compared to proself behaviour (purchase equivalent items for themselves) urged by a proself message. Furthermore, Yardley, Miller [36] showed higher levels of reported hand-washing rates than those in the control group (with or without baseline measurement). Hand-washing rates in the intervention group were higher at 4 weeks than in the control group and remained higher at 12 weeks.

Supplementary Table S5. Inter-rater agreement for MINDSPACE Coding (PABAK)

PABAK agreement between trained coder pairs for MINDSPACE contextual influencers present at least once in the included unique arms*

MINDSPACE Label	*N* where MINDSPACE contextual influencers was present across the included unique arms*	PABAK†	Confidence Intervals
Messenger	44	0.97	0.93, 1.00
Incentives	3	0.99	0.96, 1.00
Norms	16	0.96	0.91, 1.00
Defaults	22	0.78	0.67, 0.88
Salience	120	0.99	0.96, 1.00
Priming	5	0.94	0.89, 1.00
Affect	52	0.89	0.81, 0.96
Commitments	3	0.97	0.93, 1.00
Ego	43	0.87	0.79, 0.95

*140 unique included arms

†To calculate PABAK, the KAPPAETC module in Stata was used to produce the Brennan-Prediger statistic

Supplementary Note 3. Inter-rater agreement for MINDSPACE Coding

All MINDSPACE contextual influencers were identified in the papers by at least one reviewer across 140 included arms (M: 2.20; SD: 1.08; the maximum number of contextual influencers included in a study was five). The six most common MINDSPACE contextual influencers were “Messenger” (n=44), “Norms” (n=16), “Defaults” (n=22), “Salience” (n=120), “Affect” (n=52), “Ego” (n=43). “Incentives”, “Priming” and “Commitments” appeared less than ten times across the 140 arms.

Six MINDSPACE contextual influencers were identified across 38 control/ comparator arms. Ten control/ comparator arms did not include any MINDSPACE contextual influencers. 28 control/ comparator arms included at least one MINDSPACE contextual influencers (M: 1.34; SD: 1.07; the maximum number of contextual influencers included in a study was three). The three most frequent identified contextual influencers were “Messenger” (n=11), “Salience” (n=24), “Affect” (n=10). Three contextual influencers appeared less than ten times across the 28 control/ comparator arms.

Nine MINDSPACE contextual influencers were identified across 102 intervention arms (M: 2.52; SD: 0.90; the maximum number of contextual influencers included in a study was five). One intervention arm did not include MINDSPACE contextual influencers. The six most common MINDSPACE contextual influencers were “Messenger” (n=33), “Norms” (n=16), “Defaults” (n=19), “Salience” (n=96), “Affect” (n=42), “Ego” (n=43). 74 intervention arms were focused on protecting others (M: 2.60; SD: 0.89; “Messenger” (69.7%), “Norms” (68.8%), “Defaults” (73.7%), “Salience” (72.9%), “Affect” (61.9%), “Ego” (97%)), while 28 intervention arms were either self-focused or more generic.

Excellent intercoder reliability was observed across the MINDSPACE contextual influencers identified in the studies. In particular, 88.9% (n=8) with excellent agreement achieved mean PABAK scores from 0.87 to 0.99 (M: 0.94; SD: 0.04) while 11.1% (n=1) with good agreement achieved mean PABAK scores of 0.78.

Supplementary Table S6. Inter-rater agreement for BCT Coding (PABAK)

PABAK agreement between trained coder pairs for BCTs present at least once in the included unique arms*

Behaviour Change Technique (BCT) Label	*N* where BCT was present across the included unique arms*	PABAK†	Confidence Intervals
1.1 Goal setting (behaviour)	3	0.99	0.96, 1.00
1.2. Problem solving	1	0.99	0.96, 1.00
1.4. Action planning	3	0.99	0.96, 1.00
1.9. Commitment	1	1.00	1.00, 1.00
2.2. Feedback on behaviour	1	1.00	1.00, 1.00
2.3. Self-monitoring of behaviour	1	1.00	1.00, 1.00
3.1. Social support (unspecified)	1	0.82	0.73, 0.92
3.2. Social support (practical)	3	1.00	1.00, 1.00
4.1. Instruction on how to perform the behaviour	123	1.00	1.00, 1.00
4.2. Information about antecedents	1	0.99	0.96, 1.00
5.1. Information about health consequences	105	0.92	0.87, 0.99
5.2. Salience of consequences	79	0.97	0.93, 1.00
5.3. Information about social and environmental consequences	32	0.96	0.91, 1.00
5.4. Monitoring of emotional consequences	1	1.00	1.00, 1.00
6.1. Demonstration of the behaviour	6	1.00	1.00, 1.00
6.2. Social comparison	10	1.00	1.00, 1.00
6.3. Information about others' approval	6	1.00	1.00, 1.00
7.1. Prompts/cues	24	0.83	0.74, 0.92
8.1. Behavioural practice/rehearsal	1	0.99	0.96, 1.00
8.3. Habit formation	1	0.99	0.96, 1.00
9.1. Credible source	45	0.93	0.87, 0.99
10.1. Material incentive (behaviour)	2	0.97	0.93, 1.00
12.1. Restructuring the social environment	2	1.00	1.00, 1.00
12.3. Avoidance/reducing exposure to cues for the behaviour	33	0.71	0.59, 0.82
12.5. Adding objects to the environment	6	0.92	0.85, 0.98
13.1. Identification of self as role model	3	0.96	0.91, 1.00
13.2. Framing/reframing	6	0.92	0.85, 0.98
13.4. Valued self-identity	2	0.97	0.93, 1.00

*140 unique included arms

†To calculate PABAK, the KAPPAETC module in Stata was used to produce the Brennan-Prediger statistic

Supplementary Note 4. Inter-rater agreement for BCT Coding

28 out of 93 defined BCTs were identified by at least one reviewer across 140 included arms. The seven most common BCTs were "Instruction on how to perform a behaviour" (n=123), "Information about health consequences" (n=105), "Salience of consequences" (n=79), "Information about social and environmental consequences" (n=32), "Credible source" (n=45), "Prompts/cues" (n=24) and "Avoidance/reducing exposure to cues for the behaviour" (n=33). 21 BCTs appeared less than ten times across the 140 arms.

11 BCTs were identified across 38 control/ comparator arms. Ten of them did not include any BCTs. 28 control/ comparator arms included at least one BCT (M: 2.39; SD: 1.65; the maximum number of BCTs included in a study was five). The four most frequent identified BCTs were "Instruction on how to perform a behaviour" (n=27) and "Information about health consequences" (n=21), "Salience of consequences" (n=10) and "Credible source" (n=11). Seven BCTs appeared less than 10 times across the 28 control/ comparator arms.

28 BCTs were identified across 102 intervention arms (M: 4.03; SD: 1.79; the maximum number of BCTs included in a study was 17). The seven most common BCTs were "Instruction on how to perform a behaviour" (n=96), "Information about health consequences" (n=84), "Salience of consequences" (n=69), "Information about social and environmental consequences" (n=28), "Credible source" (n=34), "Prompts/cues" (n=18) and "Avoidance/reducing exposure to cues for the behaviour" (n=26). 74 intervention arms were focused on protecting others (M: 4.13; SD: 1.97), while 28 intervention arms were either self-focused or more generic.

Excellent intercoder reliability was observed across the 28 BCTs identified in the studies. In particular, 60.7% (n=17) with excellent agreement achieved mean PABAK scores from 0.83 to 0.99 (M: 0.95; SD: 0.05); 3.6% (n=1) with good agreement achieved mean PABAK scores of 0.71, while 35.7% (n = 10) achieved perfect agreement.

Supplementary Table S7. Mechanisms of Action

	Mechanism of Action (MoA) Label	*N* where MoA was present across the included studies*	Studies where MoA was present
1.	Compound MoA	1	30
2.	Mental disposition MF:0000033	5	15,16,24,26,27
3.	Behavioural intention BCIO:006016	17	15-18,20,23,24,28-34,36-38
4.	Belief MF:0000041	1	28
5.	Belief about anticipated emotion BCIO:006140	1	34
6.	Belief about consequences of behaviour BCIO:006019	1	15
7.	Belief about emotional consequences of behaviour BCIO:050218	1	36
8.	Belief about message BCIO:06141	4	18,20,22,30
9.	Belief about the credibility of a message's source BCIO:006142	2	18,27
10.	Belief about one's social environment BCIO:006145	5	16,18,26,28,29
11.	Perceived norm BCIO:006039	3	26,27,36
12.	Perceived descriptive norm BCIO:006040	1	34
13.	Normative belief BCIO:006042	1	37
14.	Belief about severity of an outcome BCIO:06030	2	32,34
15.	Belief about susceptibility to a threat BCIO:050002	1	34
16.	Belief about personal susceptibility BCIO:006031	2	17,39
17.	Belief about others' susceptibility BCIO:0050000	1	39
18.	Belief about threat BCIO:0050003	4	18,23,28,37
19.	Evaluative belief about behaviour BCIO:006147	2	34,36
20.	Belief about control over behaviour BCIO:06152	1	36
21.	Self-efficacy belief for a behaviour BCIO:006154	1	34
22.	Knowledge BCIO:006052	1	30
23.	Willingness to comply BCIO:006059	2	18,28
24.	Emotion process MFOEM:000001	4	17,18,31,39
25.	Anxiety MFOEM:000028	2	16,26
26.	Belief about responsibility to act	1	22
27.	Behaviour Decision BCIO:006048	1	16
28.	Need for social relatedness BCIO:006068	1	18
29.	Belief about the personal desirability of consequences of behaviour BCIO:006149	1	37
30.	Motivation BCIO:006133	1	39

*24 included studies

Supplementary Note 5. Mechanisms of Action

A mean of 3.48 MoAs (SD = 2.02) was identified in those papers that measured at least one MoA (the maximum number of MoAs identified in a study was nine). The coding procedure was performed as detailed by the Ontology developers. This ensured that the lead researcher´s coding was consistent with the rationale of the Ontology (https://osf.io/um7w6). A total amount of 30 MoAs was coded at least once among the papers. The Compound MoA was coded because it was a composite of different MoAs averaged in a single measure. The most common MoA was “Behavioural intention”, while less frequently to appear were the MoAs “Belief about one's social environment” and “Mental disposition”. All the MoAs identified in the articles were measured with self-report measures.

Supplementary Table S8. Positively affected behaviour-related outcomes by protect others messages

Outcomes	Study	Effective Message	MoAs applied	BCTs applied	Contextual influencers applied
Social distancing	[15] Study 1 & 2	Reframing Bravery message	Belief about consequences of behaviour; Behavioural intention	4.1, 13.2, 5.1, 5.2	Default, Salience, Ego
		Other-regarding, Linear Cooperation message		4.1, 5.1, 5.2, 6.2	Norms, Default, Salience, Affect
	[16]	altruistic goal-orientation message	Behavioural intention	4.1, 9.1, 12.3	Messenger, Ego
	[22] Study 1, 2 & 3	prosocial message	Belief about message	4.1, 5.1	Salience
	[20] Study 1, 2 & 3	other-focused message	Belief about message	4.1, 5.1, 5.2, 9.1	Messenger, Salience, Ego
		Combined message		4.1, 5.1, 5.2, 9.1	Messenger, Salience, Affect, Ego
	[33]	message framed with respect to consequences for one's family	Behavioural intention	5.1, 5.2	Salience, Affect
	[39] Study 3	information + empathy	Emotion process	5.2, 5.3, 9.1, 12.3	Salience, Affect
Mask wearing	[15]	Reframing Bravery message	Behavioural intention	4.1, 13.2, 5.1, 5.2	Default, Salience, Ego
		Other-regarding, Linear Cooperation message		4.1, 5.1, 5.2, 6.2	Norms, Default, Salience, Affect
	[17]	Your community message	Behavioural intention	5.1, 5.2, 4.1	Salience, Ego
	[39]	empathy message	Motivation; Emotion process	5.1, 5.2	Salience, Affect
Hand washing	[16]	altruistic goal-orientation message	Behavioural intention	4.1, 9.1, 12.3	Messenger, Ego
	[34]	High-threat/coping message	Behavioural intention	1.1, 1.4, 4.1, 5.1, 5.2, 5.3, 9.1, 12.1, 12.3	Messenger, Default, Salience, Commitment, Ego
		Low-threat coping message		1.1, 1.4, 4.1, 5.1, 9.1, 12.1, 12.3	Messenger, Default, Salience, Commitment
		High threat message		5.1, 5.2, 5.3	Salience, Ego
	[36]	Web-based intervention with tailored motivational messages	Evaluative belief about behaviour; Belief about control over behaviour; Behavioural intention	1.1, 1.2, 1.4, 1.9, 2.2, 2.3, 3.1, 4.1, 4.2, 5.1, 5.2, 6.3, 7.1, 8.1, 8.3, 9.1, 12.5	Messenger, Norms, Default, Salience, Commitment
Diverse behavioural intentions	[22] Study 1, 2 & 3	prosocial message	Belief about responsibility to act	4.1, 5.1	Salience
	[18]	other-focused messages	Belief about message; Behavioural intention; Willingness to comply	4.1, 5.1, 5.2, 9.1	Messenger, Salience, Ego
	[37] Study 1 & 2	Public message	Behavioural intention	5.1, 5.2, 5.3, 4.1	Salience, Ego
		personal + public message		5.1, 5.2, 5.3, 4.1	Salience, Affect, Ego
		family message		5.1, 5.2, 4.1	Salience, Affect
	[24] Study 1 & 2	compassion and empathy message	Behavioural intention	5.1, 5.2, 4.1	Salience, Ego
		principle of reciprocity message		5.1, 5.2, 4.1, 6.3	Norms, Salience, Ego
	[23] Study 1 & 2	public message	Behavioural intention	5.1, 5.2, 5.3, 4.1	Salience, Ego
		personal + public message		5.1, 5.2, 5.3, 4.1	Salience, Affect, Ego
	[38]	gain-framed altruistic message	Behavioural intention	4.1, 5.1, 5.2, 5.3, 12.3, 13.2	Salience, Affect
Actual Behaviours	[19]	patient-consequences sign	n/a	4.1, 5.1, 7.1, 12.5	Default, Salience, Priming
	[21]	Prosocial message	n/a	3.2, 4.1, 5.1, 10.1	Incentives, Salience, Ego
	[36]	Web-based intervention with tailored motivational messages	Evaluative belief about behaviour; Belief about control over behaviour; Behavioural intention	1.1, 1.2, 1.4, 1.9, 2.2, 2.3, 3.1, 4.1, 4.2, 5.1, 5.2, 6.3, 7.1, 8.1, 8.3, 9.1, 12.5	Messenger, Norms, Default, Salience, Commitment

** Each number represents a behaviour change techniques cluster, and the technique within that number (Michie S, Richardson M, Johnston M, et al. Annals of behavioral medicine 2013; 46(1): 81-95[7]).*

Supplementary Table S8. NMA effect estimates on social distancing intentions

Comparison	Effect estimates: SMD	95% CrI
Prosocial message focused on public	0.07	0.01, 0.13
Prosocial message focused on loved ones	0.10	0.04, 0.16
Self-focused message	-*	-
Model fit and heterogeneity	Between-study SD	95% CrI
	0.03	0.01, 0.11

*Total residual deviance: Mean 27.38 from 27 datapoints; * Insufficient data to estimate*

Supplementary Figure S2. Network Diagram for social distancing intentions

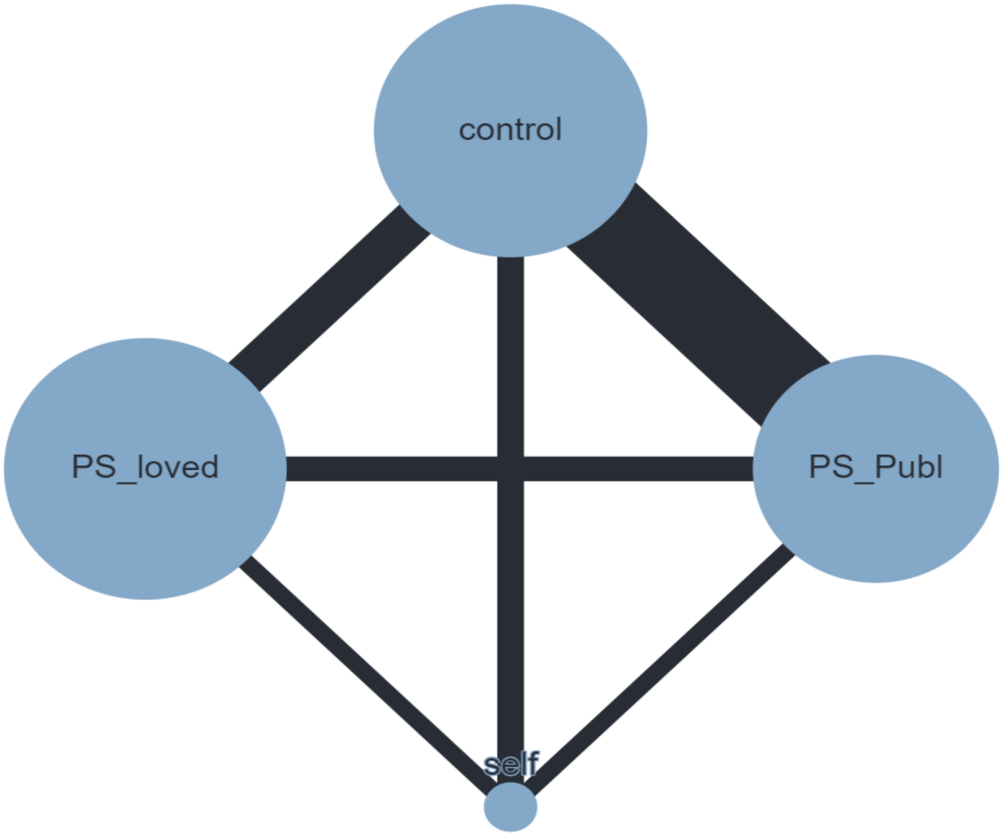

PS_Love: prosocial messages focused on loved ones, PS_Publ: prosocial messages focused on public, sel: self-focused messages

Supplementary Table S9. NMA effect estimates on mask wearing intentions

Comparison	Effect estimates: SMD	95% CrI
Prosocial message focused on public	0.16	0.04, 0.30
Prosocial message focused on loved ones	0.06	-0.04, 0.17
Self-focused message	0.06	-0.07, 0.20
Model fit and heterogeneity	Between-study SD	95% CrI
	0.05	0.01, 0.22

Total residual deviance: Mean 10.71 from 11 datapoints

Supplementary Figure S3. Network diagram for mask wearing intentions

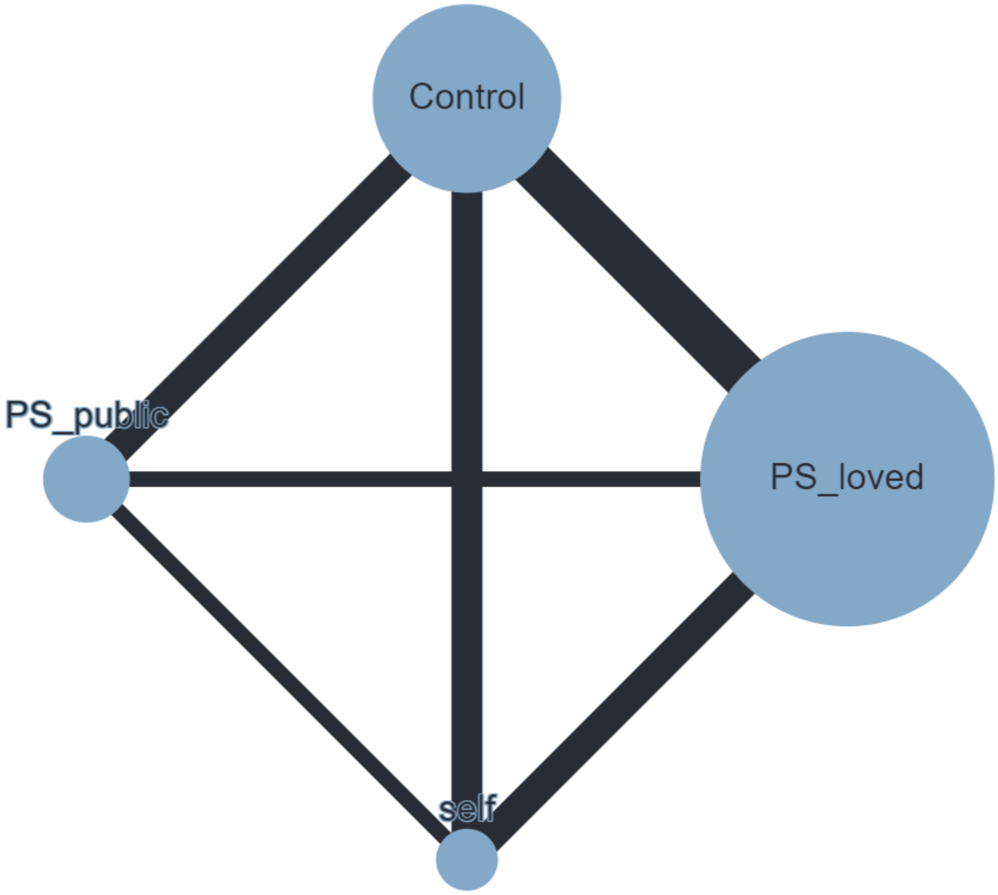

PS_Love: prosocial messages focused on loved ones, PS_Publ: prosocial messages focused on public, sel: self-focused messages

Supplementary Table S10. NMA effect estimates on handwashing intentions

Comparison	Effect estimates: SMD	95% CrI
Prosocial message focused on public	0.10	-0.20, 0.39
Prosocial message focused on loved ones	0.20	-0.15, 0.52
Self-focused message	0.08	-0.37, 0.52
Model fit and heterogeneity	Between-study SD	95% CrI
	0.28	0.14, 0.60

Total residual deviance: Mean 19.62 from 18 datapoints

Supplementary Figure S4. Network diagram for handwashing intentions

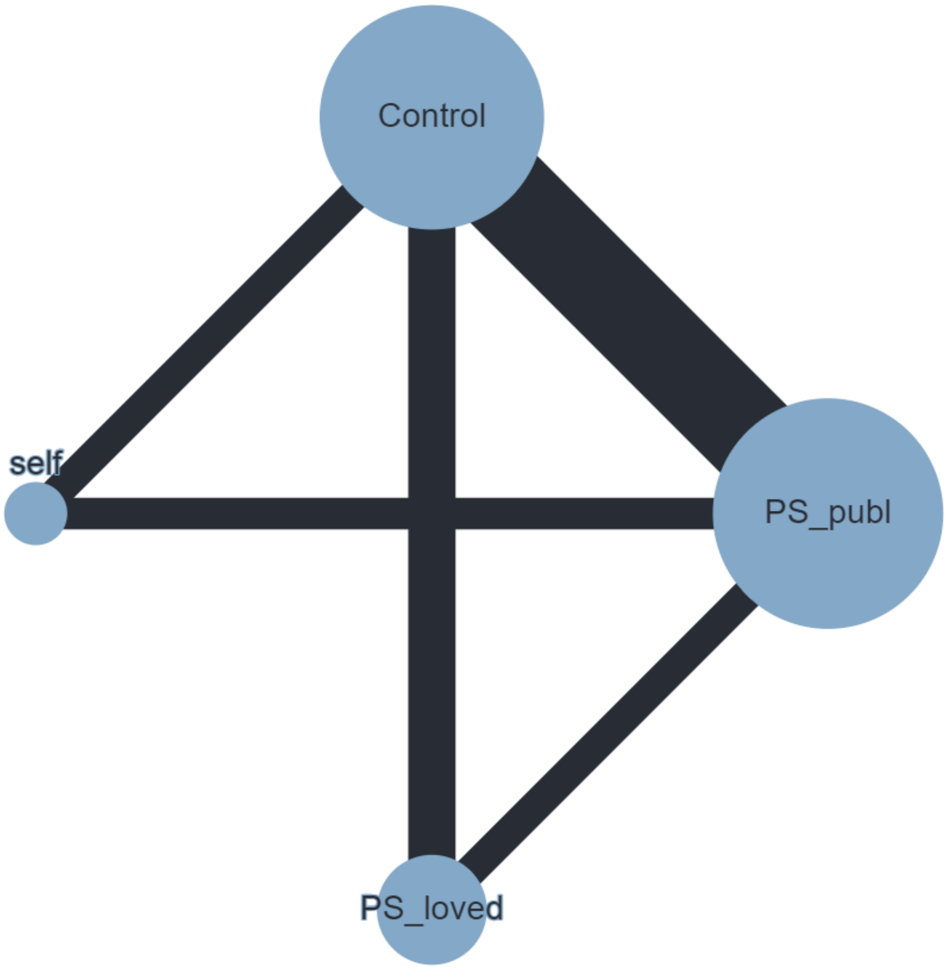

PS_Love: prosocial messages focused on loved ones, PS_Publ: prosocial messages focused on public, sel: self-focused messages

Supplementary Table S11. NMA effect estimates on diverse-behavioural intentions

Comparison	Effect estimates: SMD	95% CrI
Prosocial message focused on public	0.10	0.01, 0.21
Prosocial message focused on loved ones	0.17	0.04, 0.31
Self-focused message	0.10	-0.01, 0.23
Model fit and heterogeneity	Between-study SD	95% CrI
	0.11	0.07, 0.19

Total residual deviance: Mean 21.34 from 24 datapoints

Supplementary Figure S5. Network diagram for diverse-behavioural intentions

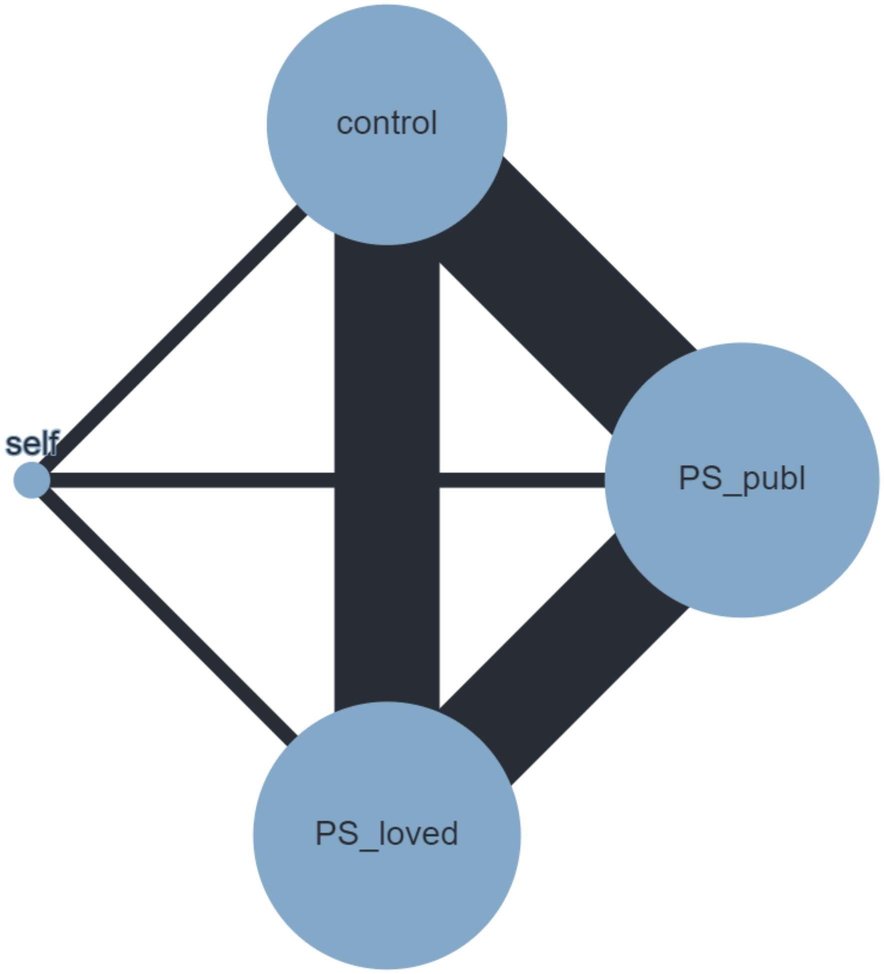

PS_Love: prosocial messages focused on loved ones, PS_Publ: prosocial messages focused on public, sel: self-focused messages

Supplementary Table S12. CNMA effect estimates of MINDSPACE contextual influencers on personal protective behavioural intentions (self-focused messages)

Comparison	Effect estimates: SMD	95% CrI
Salience	-0.03	-4.84, 2.60
Affect	0.10	-2.53, 4.92
Model fit and heterogeneity	Between-study SD	95% CrI
	0.10	0.02, 0.21

Total residual deviance: Mean 20.96 from 20 datapoints

Supplementary Note 6. CNMAs of MINDSPACE for interventions using self-focused messages

The CNMA model with Mindspace contextual influencers had a reasonable fit (total residual deviance=20.96 from 20 data points) and relatively low heterogeneity (SD= 0.10, 95% CrI 0.02 to 0.21). There was a small increase in personal protective behavioural intentions for interventions that incorporated the "affect" (d= 0.10, 95% CrI 2.53 to 4.92) compared to interventions that did not include this contextual influencer.

Supplementary Table S13. ***CNMA effect estimates of BCTs on personal protective behavioural intentions (self-focused messages)***

Comparison	Effect estimates: SMD	95% CrI
4.1. Instruction on how to perform the behaviour	-0.02	-3.03, 1.48
5.1. Information about health consequences	0.05	-1.47, 3.04
5.2. Salience of consequences	0.07	-0.19, 0.36
6.2. Social comparison	-0.04	-0.28, 0.21
Model fit and heterogeneity	Between-study SD	95% CrI
	0.11	0.03, 0.27

Total residual deviance: Mean 20.71 from 20 datapoints.

Supplementary Note 7. CNMAs of BCTs for interventions using self-focused messages

The CNMA model had a reasonable fit (total residual deviance=20.71 from 20 data points) and relatively low heterogeneity (SD= 0.11, 95% CrI 0.03 to 0.27). There was a small increase in personal protective behavioural intentions for interventions that incorporated the "information about health consequences" (d=0.05; 95% Crl -1.47 to 3.04) and the "salience of consequences" (d=0.07; 95% Crl -0.19 to 0.36) compared to interventions that did not include these BCTs.

Supplementary Figure S6. Certainty of the evidence: Personal protective behavioural intentions

Comparison	Number of Studies	Within-study bias	Reporting bias	Indirectness	Imprecision	Heterogeneity	Incoherence	Confidence rating	Reason(s) for downgrading
				Mixed evidence					
No prosocial message (self-focused) vs Prosocial message Public	10	Some concerns ☑	Low risk	No concerns	No concerns	No concerns	No concerns	Moderate	| Within-study bias |
No prosocial message (self-focused) vs Prosocial message loved ones	5	Some concerns ☑	Low risk	No concerns	No concerns	No concerns	No concerns	Moderate	| Within-study bias |
No prosocial message (self-focused) vs control	12	Some concerns ☑	Low risk	No concerns	No concerns	No concerns	Some concerns ☑	Low	| Within-study bias | Incoherence |
Prosocial message Public vs Prosocial message loved ones	9	Some concerns ☑	Low risk	No concerns	No concerns	No concerns	No concerns	Moderate	| Within-study bias |
Prosocial message Public vs control	26	Some concerns ☑	Low risk	No concerns	No concerns	Some concerns ☑	No concerns	Low	| Within-study bias | Heterogeneity |
Prosocial message loved ones vs control	16	Some concerns ☑	Low risk	No concerns	No concerns	Some concerns ☑	No concerns	Low	| Within-study bias | Heterogeneity |

The low confidence rating suggests a lack of confidence in the evidence presented. In our analysis comparing prosocial messages (both focused on the public and loved ones) versus the control group, the low quality of evidence is primarily attributed to within-study bias and heterogeneity. These issues may be anticipated due to the limited number of included studies and the diverse range of outcomes assessed. Therefore, the results should be interpreted with caution

Supplementary Figure S7. Certainty of the evidence: Social distancing intentions

Comparison	Number of Studies	Within-study bias	Reporting bias	Indirectness	Imprecision	Heterogeneity	Incoherence	Confidence rating	Reason(s) for downgrading
			Mixed evidence						
No prosocial message (self-focused) vs Prosocial message Public	2	Some concerns ☑	Low risk	No concerns	Some concerns ☑	No concerns	No concerns	Low	| Within-study bias | Imprecision |
No prosocial message (self-focused) vs Prosocial message loved ones	2	Some concerns ☑	Low risk	No concerns	No concerns	No concerns	No concerns	Moderate	| Within-study bias |
No prosocial message (self-focused) vs control	3	Some concerns ☑	Low risk	No concerns	No concerns	No concerns	No concerns	Moderate	| Within-study bias |
Prosocial message Public vs Prosocial message loved ones	3	Some concerns ☑	Low risk	No concerns	No concerns	Some concerns ☑	No concerns	Low	| Within-study bias | Heterogeneity |
Prosocial message Public vs control	10	Some concerns ☑	Low risk	No concerns	No concerns	No concerns	No concerns	Moderate	| Within-study bias |
Prosocial message loved ones vs control	6	Some concerns ☑	Low risk	No concerns	No concerns	No concerns	No concerns	Moderate	| Within-study bias |

The moderate confidence rating suggests a likelihood that the actual effect is similar to the NMA estimates but could differ substantially. In our analysis comparing prosocial messages (both focused on the public and loved ones) versus the control group, the moderate quality of evidence is primarily attributed to within-study bias.

Supplementary Figure S8. Certainty of the evidence: Mask wearing intentions

Comparison	Number of Studies	Within-study bias	Reporting bias	Indirectness	Imprecision	Heterogeneity	Incoherence	Confidence rating	Reason(s) for downgrading
			Mixed evidence						
No prosocial message (self-focused) vs Prosocial message Public	1	Some concerns ☑	Low risk	No concerns	No concerns	No concerns	No concerns	Moderate	| Within-study bias |
No prosocial message (self-focused) vs Prosocial message loved ones	2	Some concerns ☑	Low risk	No concerns	No concerns	No concerns	No concerns	Moderate	| Within-study bias |
No prosocial message (self-focused) vs control	2	Some concerns ☑	Low risk	No concerns	No concerns	No concerns	No concerns	Moderate	| Within-study bias |
Prosocial message Public vs Prosocial message loved ones	1	Some concerns ☑	Low risk	No concerns	No concerns	No concerns	No concerns	Moderate	| Within-study bias |
Prosocial message Public vs control	2	Some concerns ☑	Low risk	No concerns	No concerns	No concerns	No concerns	Moderate	| Within-study bias |
Prosocial message loved ones vs control	3	Some concerns ☑	Low risk	No concerns	No concerns	No concerns	No concerns	Moderate	| Within-study bias |

The moderate confidence rating suggests a likelihood that the actual effect is similar to the NMA estimates but could differ substantially. In our analysis comparing prosocial messages (both focused on the public and loved ones) versus the control group, the moderate quality of evidence is primarily attributed to within-study bias.

Supplementary Figure S9. Certainty of the evidence: Handwashing intentions

Comparison	Number of Studies	Within-study bias	Reporting bias	Indirectness	Imprecision	Heterogeneity	Incoherence	Confidence rating	Reason(s) for downgrading
Mixed evidence									
No prosocial message (self-focused) vs Prosocial message Public	2	Some concerns ☑	Low risk	No concerns	Major concerns ☐	No concerns	No concerns	Very low	| Within-study bias |
No prosocial message (self-focused) vs control	2	Some concerns ☑	Low risk	No concerns	Major concerns ☐	No concerns	No concerns	Very low	| Within-study bias |
Prosocial message Public vs Prosocial message loved ones	2	Some concerns ☑	Low risk	No concerns	Some concerns ☑	Some concerns ☑	Some concerns ☑	Low	| Within-study bias | Imprecision | Heterogeneity | Incoherence |
Prosocial message Public vs control	5	Some concerns ☑	Low risk	No concerns	Some concerns ☑	Some concerns ☑	Some concerns ☑	Low	| Within-study bias | Imprecision | Heterogeneity | Incoherence |
Prosocial message loved ones vs control	4	Some concerns ☑	Low risk	No concerns	No concerns	Some concerns ☑	No concerns	Low	| Within-study bias | Heterogeneity |
Indirect evidence									
No prosocial message (self-focused) vs Prosocial message loved ones	--	Some concerns ☑	Low risk	No concerns	Some concerns ☑	Some concerns ☑	No concerns	Low	| Within-study bias | Imprecision | Heterogeneity |

The low confidence rating suggests a lack of confidence in the evidence presented. In our analysis comparing prosocial messages focus on loved ones versus the control group, the low quality of evidence is primarily attributed to within-study bias and heterogeneity, while comparing prosocial messages focus on public versus the control group, the low quality of evidence is primarily attributed to within-study bias, imprecision, heterogeneity and incoherence.

Supplementary Figure S10. Certainty of the evidence: diverse-behavioural intentions

Comparison	Number of Studies	Within-study bias	Reporting bias	Indirectness	Imprecision	Heterogeneity	Incoherence	Confidence rating	Reason(s) for downgrading
				Mixed evidence					
control vs loved ones	3	Some concerns ☑	Low risk	No concerns	No concerns	Major concerns ☑	No concerns	Very low	| Within-study bias | Heterogeneity |
control vs public	9	Some concerns ☑	Low risk	No concerns	Some concerns ☑	Some concerns ☑	Major concerns ☑	Very low	| Within-study bias | Imprecision | Heterogeneity | Incoherence |
control vs self focused	5	Some concerns ☑	Low risk	No concerns	Major concerns ☑	No concerns	No concerns	Very low	| Within-study bias | Imprecision |
loved ones vs public	3	Some concerns ☑	Low risk	No concerns	Some concerns ☑	Some concerns ☑	No concerns	Low	| Within-study bias | Imprecision | Heterogeneity |
loved ones vs self focused	1	Some concerns ☑	Low risk	No concerns	Major concerns ☑	No concerns	No concerns	Very low	| Within-study bias | Imprecision |
public vs self focused	5	Some concerns ☑	Low risk	No concerns	Major concerns ☑	No concerns	No concerns	Very low	| Within-study bias | Imprecision |

The very low confidence rating suggests a high likelihood of substantial differences. In our analysis comparing prosocial messages focus on loved ones versus the control group, the very low quality of evidence is primarily attributed to within-study bias and heterogeneity, while comparing prosocial messages focus on public versus the control group, the very low quality of evidence is primarily attributed to within-study bias, imprecision, heterogeneity and incoherence.

Supplementary References

1. Penner LA, Dovidio JF, Piliavin JA, Schroeder DA. Prosocial behavior: Multilevel perspectives. *Annu Rev Psychol* 2005; **56**: 365-92.
2. Smith HS, Blumenthal-Barby JS, Chatterjee R, et al. A Review of the MINDSPACE Framework for Nudging Health Promotion During Early Stages of the COVID-19 Pandemic. *Popul Health Manag* 2022; **25**(4): 487-500.
3. Dolan P, Hallsworth M, Halpern D, King D, Metcalfe R, Vlaev I. Influencing behaviour: The mindspace way. *Journal of Economic Psychology* 2012; **33**(1): 264-77.
4. Thaler RH, Sunstein CR. Nudge: Improving decisions about health, wealth, and happiness: Penguin; 2009.
5. Vlaev I, King D, Dolan P, Darzi A. The theory and practice of "nudging": changing health behaviors. *Public Administration Review* 2016; **76**(4): 550-61.
6. O'Sullivan M, Ryan C, Downey DG, Hughes CM. A change in behaviour: getting the balance right for research and policy. *Int J Clin Pharm* 2016; **38**(5): 1027-31.
7. Michie S, Richardson M, Johnston M, et al. The behavior change technique taxonomy (v1) of 93 hierarchically clustered techniques: building an international consensus for the reporting of behavior change interventions. *Annals of behavioral medicine* 2013; **46**(1): 81-95.
8. Michie S, Atkins L, West R. The behaviour change wheel: a guide to designing interventions. Great Britain Silverback Publishing; 2014.
9. Michie S, Van Stralen MM, West R. The behaviour change wheel: a new method for characterising and designing behaviour change interventions. *Implementation science* 2011; **6**(1): 42.
10. Michie S, Wood CE, Johnston M, Abraham C, Francis J, Hardeman W. Behaviour change techniques: the development and evaluation of a taxonomic method for reporting and describing behaviour change interventions (a suite of five studies involving consensus methods, randomised controlled trials and analysis of qualitative data). *Health Technology Assessment* 2015; **19**(99): 1–188.
11. Armitage CJ, Conner M, Prestwich A, et al. Investigating which behaviour change techniques work for whom in which contexts delivered by what means: Proposal for an international collaboratory of Centres for Understanding Behaviour Change (CUBiC). *British Journal of Health Psychology* 2021; **26**(1): 1-14.
12. Michie S, Wood CE, Johnston M, Abraham C, Francis JJ, Hardeman W. Training to code intervention descriptions using Behaviour Change Technique Taxonomy version 1 (study 3). Behaviour change techniques: the development and evaluation of a taxonomic method for reporting and describing behaviour change interventions (a suite of five studies involving consensus methods, randomised controlled trials and analysis of qualitative data): NIHR Journals Library; 2015.
13. Michie S, Thomas J, Johnston M, et al. The Human Behaviour-Change Project: harnessing the power of artificial intelligence and machine learning for evidence synthesis and interpretation. *Implement Sci* 2017; **12**(1): 121.
14. Michie S, West R, Finnerty AN, et al. Representation of behaviour change interventions and their evaluation: Development of the Upper Level of the Behaviour Change Intervention Ontology. *Wellcome open research* 2020; **5**: 123.
15. Bokemper SE, Huber GA, James EK, Gerber AS, Omer SB. Testing persuasive messaging to encourage COVID-19 risk reduction. *PLoS One* 2022; **17**(3): e0264782.
16. Browning A, Moss ME, Berkman E. Leveraging Evidence-Based Messaging to Prevent the Spread of COVID-19. 2021.
17. Capraro V, Barcelo H. The effect of messaging and gender on intentions to wear a face covering to slow down COVID-19 transmission. *Journal of Behavioral Economics for Policy* 2020; **4**(2): 45-55.
18. Gillman AS, Iles IA, Klein WM, Ferrer RA. Increasing Receptivity to COVID-19 Public Health Messages with Self-Affirmation and Self vs. Other Framing. *Health Communication* 2022; **38**(9): 1942–53.
19. Grant AM, Hofmann DA. It's not all about me: Motivating hand hygiene among health care professionals by focusing on patients. *Psychological science* 2011; **22**(12): 1494-9.
20. Luttrell A, Petty RE. Evaluations of self-focused versus other-focused arguments for social distancing: An extension of moral matching effects. *Social Psychological Personality Science* 2021; **12**(6): 946-54.
21. Varma MM, Chen D, Lin X, Aknin LB, Hu X. Prosocial behavior promotes positive emotion during the COVID-19 pandemic. *Emotion* 2022; **23**(2): 538–53.
22. Ceylan M, Hayran C. Message Framing Effects on Individuals' Social Distancing and Helping Behavior During the COVID-19 Pandemic. *Front Psychol* 2021; **12**: 579164.
23. Jordan J, Yoeli E, Rand D. Don't get it or don't spread it: Comparing self-interested versus prosocial motivations for COVID-19 prevention behaviors. *Scientific reports* 2021; **11**(1): 1-17.

24. Pink S, Stagnaro M, Chu J, Mernyk J, Voelkel JG, Willer R. The effects of short messages encouraging prevention behaviors early in the COVID-19 pandemic. *PLoS One* 2023; **18**(4): e0284354.
25. Banker S, Park J. Evaluating prosocial COVID-19 messaging frames: Evidence from a field study on Facebook. *Judgment Decision Making* 2020; **15**(6): 1037-43.
26. Barari S, Caria S, Davola A, et al. Evaluating COVID-19 public health messaging in Italy: Self-reported compliance and growing mental health concerns. MedRxiv; 2020.
27. Everett JA, Colombatto C, Chituc V, Brady WJ, Crockett M. The effectiveness of moral messages on public health behavioral intentions during the COVID-19 pandemic. 2020.
28. Favero N, Pedersen MJ. How to encourage "Togetherness by Keeping Apart" amid COVID-19? The ineffectiveness of prosocial and empathy appeals. *Journal of Behavioral Public Administration* 2020; **3**(1): 1-18.
29. Frias-Navarro D, Pascual-Soler M, Berrios-Riquelme J, Gomez-Frias R, Caamaño-Rocha L. COVID–19. Effect of Moral Messages to Persuade the Population to Stay at Home in Spain, Chile, and Colombia. *The Spanish Journal of Psychology* 2021; **24**: e42.
30. Hacquin A-S, Mercier H, Chevallier C. Improving preventive health behaviors in the COVID-19 crisis: a messaging intervention in a large nationally representative sample. 2020.
31. Heffner J, Vives ML, FeldmanHall O. Emotional responses to prosocial messages increase willingness to self-isolate during the COVID-19 pandemic. *Pers Individ Dif* 2021; **170**: 110420.
32. Utych SM, Fowler L. Age-based messaging strategies for communication about COVID-19. *Journal of Behavioral Public Administration* 2020; **3**(1): 1-14.
33. Falco P, Zaccagni S. Promoting social distancing in a pandemic: Beyond good intentions. *PLoS One* 2021; **16**(12): e0260457.
34. Miller S, Yardley L, Little P, team P. Development of an intervention to reduce transmission of respiratory infections and pandemic flu: measuring and predicting hand-washing intentions. *Psychol Health Med* 2012; **17**(1): 59-81.
35. Pfattheicher S, Nockur L, Böhm R, Sassenrath C, Petersen MB. The emotional path to action: Empathy promotes physical distancing during the COVID-19 pandemic. 2020.
36. Yardley L, Miller S, Schlotz W, Little P. Evaluation of a Web-based intervention to promote hand hygiene: exploratory randomized controlled trial. *Journal of medical Internet research* 2011; **13**(4): e1963.
37. Miyajima T, Murakami F. Self-interested framed and prosocially framed messaging can equally promote COVID-19 prevention intention: A replication and extension of Jordan et al.'s study (2020) in the Japanese context. *Frontiers in psychology* 2021; **12**: 1341.
38. Sasaki S, Kurokawa H, Ohtake F. Effective but fragile? Responses to repeated nudge-based messages for preventing the spread of COVID-19 infection. *The Japanese Economic Review* 2021; **72**(3): 371-408.
39. Pfattheicher S, Nockur L, Böhm R, Sassenrath C, Petersen MB. The Emotional Path to Action: Empathy Promotes Physical Distancing and Wearing of Face Masks During the COVID-19 Pandemic. *Psychological Science* 2020; **31**(11): 1363-73.
