# Supplementary material for: Motivational effectiveness of prosocial public health messaging to reduce respiratory infection risk: a systematic review and meta-analysis
Source: Commun Med (Lond). 2025 Dec 24;6:42. doi: 10.1038/s43856-025-01296-6 (PMC12820126; doi:10.1038/s43856-025-01296-6)
Supplement: Supplementary file 3 — Description of Additional Supplementary files [file 43856_2025_1296_MOESM3_ESM.pdf]

Description of Additional Supplementary Files

File name: Supplementary Data 1

Description: Mechanism of Actions

File name: Supplementary Data 2

Description: General Characteristics of the included studies

File name: Supplementary Data 3

Description: Intervention characteristics

File name: Supplementary Data 4

Description: Risk of Bias assessment of included studies

File name: Supplementary Data 5

Description: Effective Ratios of MINDSPACE and behaviour change techniques

File name: Supplementary Data 6

Description: Data of Network meta-analysis
